# CLINTERVENTIONAL protocol: a randomized controlled trial to evaluate clinical consultations and audiovisual tools for interventional radiology

**DOI:** 10.1186/s41747-024-00545-y

**Published:** 2025-01-15

**Authors:** Pedro Blas García Jurado, Juan José Espejo Herrero, María Sagrario Lombardo Galera, María Eugenia Pérez Montilla, Sara Barranco Acosta, José García-Revillo, Pilar Font Ugalde, Marina Álvarez Benito

**Affiliations:** 1 Maimónides Biomedical Research Institute of Córdoba (IMIBIC), Córdoba, Spain; 2 University of Córdoba, Córdoba, Spain; 3 Department of Radiology, Reina Sofía University Hospital, Menéndez Pidal Avenue s/n, 14004 Córdoba, Spain

**Keywords:** Audiovisual aids, Communication, Patient satisfaction, Physician-patient relations, Radiology (interventional)

## Abstract

**Abstract:**

Interventional radiology (IR) has evolved rapidly, but the clinical integration of interventional radiologists has not kept pace with technical advancements. This trial will address a gap in the literature by providing a robust investigation into specific measures for enhancing the clinical role of interventional radiologists, with potential implications for improving patient experiences and outcomes. The single-center randomized controlled trial will include 428 patients undergoing IR procedures. The control group will receive information about the procedure from the ordering physician, while the experimental group will have an additional consultation with an interventional radiologist and be shown procedure-specific explanatory videos. The primary outcomes are patients’ knowledge, satisfaction with the information and communication, and anxiety. Data collection will involve specific questionnaires and scales. This trial is designed to investigate the importance of proactive clinical roles in patient care within IR. The study explores the potential of consultations and audiovisual tools, highlighting their role in educating patients about procedures. The results may help foster a more widespread acceptance of clinical responsibilities in IR and underscore the pivotal role of audiovisual aids in patient education and satisfaction.

**Trial registration:**

NCT05461482 at clinicaltrials.gov.

**Relevance statement:**

This randomized controlled trial will assess the impact of clinical consultations and explanatory audiovisual tools on patient understanding, satisfaction, and anxiety in interventional radiology. The findings could help establish a more proactive clinical role for interventional radiologists and improve the overall quality of patient-centered care.

**Key Points:**

- We describe the protocol of an interventional radiology randomized clinical trial.
- The control group will receive procedure information from the referring physician and the experimental group receives additional consultation with interventionalists and views a video.
- Knowledge, satisfaction with information, and patient anxiety will be evaluated.
- This study will provide insights about the benefits of consultations and videos in interventional radiology.

**Graphical Abstract:**

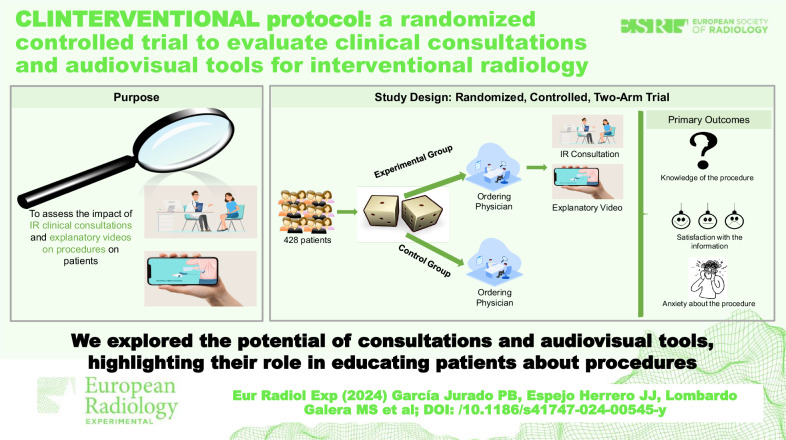

## Background

Interventional radiology (IR) has developed rapidly since its inception in the 1960s and has now become an essential part of modern medicine [1, 2]. However, the swift development of IR techniques has not been accompanied by the emergence of a clinical role for interventional radiologists [3–5]. The movement of IR toward a more clinical role focused on patient care (before, during, and after interventions) has been slow and heterogeneous due to various interrelated factors [3]: the disparate recognition of IR as an area of knowledge differentiated from other specialties and subspecialties and the consequent lack of standardization of official IR training [6]; the historical origins of IR in traditional radiology [7, 8]; the lack of training of interventional radiologists in clinical skills [3]; and the lack of infrastructure and professional resources [8].

IR scientific societies and experts agree that the cornerstone for the consolidation of IR is its transition to an active clinical role. However, the existing bibliography about the impact of specific measures for developing this clinical role in IR is limited [9–11].

This randomized controlled trial (RCT) intends to evaluate whether the implementation of IR clinical consultations and the use of videos to explain the procedures have an impact on patients’ understanding of the procedures, their satisfaction with the information provided, and the anxiety they experience related to the procedures. To the best of our knowledge, this is the first RCT investigating this issue.

## Objectives and study design

The CLINTERVENTIONAL study is a single-center, two-arm RCT, registered at clinicaltrials.gov (NCT05461482). In the control arm, patients will be informed about the IR procedure by the ordering physician, which is the usual process in the hospital. In the experimental arm, patients will have an additional consultation with an interventional radiologist and will be shown an explanatory video about the procedure. This trial is independent of industry funding. It has been initiated and conducted with resources from the Maimónides Biomedical Research Institute of Córdoba and Reina Sofía University Hospital (Córdoba, Spain). The study protocol, informed consent form template, and other requested documents regarding both the scientific content and compliance with applicable regulations regarding research and human subjects have been reviewed and approved by the Research Ethics Committee of Córdoba. The trial will be conducted in accordance with the Declaration of Helsinki.

Participants will receive a clinical trial information sheet and must provide written informed consent (Appendix 1: Clinical trial information sheet and sample informed consent form). The allocation of patients will be done using random number tables with a 1:1 allocation ratio. The participants, data analysts, and outcome evaluators will be blinded to the arm assignment.

The main objective is to assess and compare both groups at baseline and after the IR procedure in terms of patients’ level of knowledge and understanding of the IR procedures, patients’ satisfaction with the information provided and the method of communication, and patients’ anxiety

There are four secondary objectives. The first is to analyze changes within each group in patients’ level of knowledge and understanding of the IR procedures, patients’ satisfaction with the information provided and the method of communication, and patients’ anxiety at the beginning of the RCT and at the end. The second is to analyze and compare the duration of the intervention, the patients’ perceived pain intensity during the procedure, the level of tolerance for the intervention, and the patients’ satisfaction level with the intervention performed. The third is to evaluate the association of different variables (demographics, personal history, assigned arm, type of IR procedure, anxiety related to the IR procedure, knowledge and understanding of the IR procedure, type of anesthesia, and degree of sedation) with the intensity of pain during the intervention, tolerance of the procedure, and satisfaction with the intervention. The fourth is to evaluate explanatory videos as an educational tool.

### Study cohort and outcome measures

Patients who are planned to undergo an IR procedure at the Reina Sofía University Hospital will be candidates for participation in the trial. Full inclusion and exclusion criteria are provided in Table 1.

**Table 1:** Eligibility criteria

Inclusion criteria	Exclusion criteria
Patients undergoing one of the following vascular or non-vascular procedures: • Endovascular recanalization; • Placement of tunneled central venous catheters; • Fistulograms and endovascular treatment of hemodialysis fistulas; • Endovascular embolization; • Percutaneous biopsies; • Percutaneous drainage of collections; • Percutaneous transhepatic biliary drainage; • Percutaneous nephrostomies.	• Patients under 18 years of age. • Patients requiring the involvement of a third party for informed consent. • Pregnant patients. • Patients undergoing urgent interventional procedures. • Patients with allergies or intolerance to mepivacaine, tramadol, midazolam, or fentanyl. • Patients who do not understand and speak Spanish proficiently. • Patients with deafness or blindness.

**Fig. 1:**
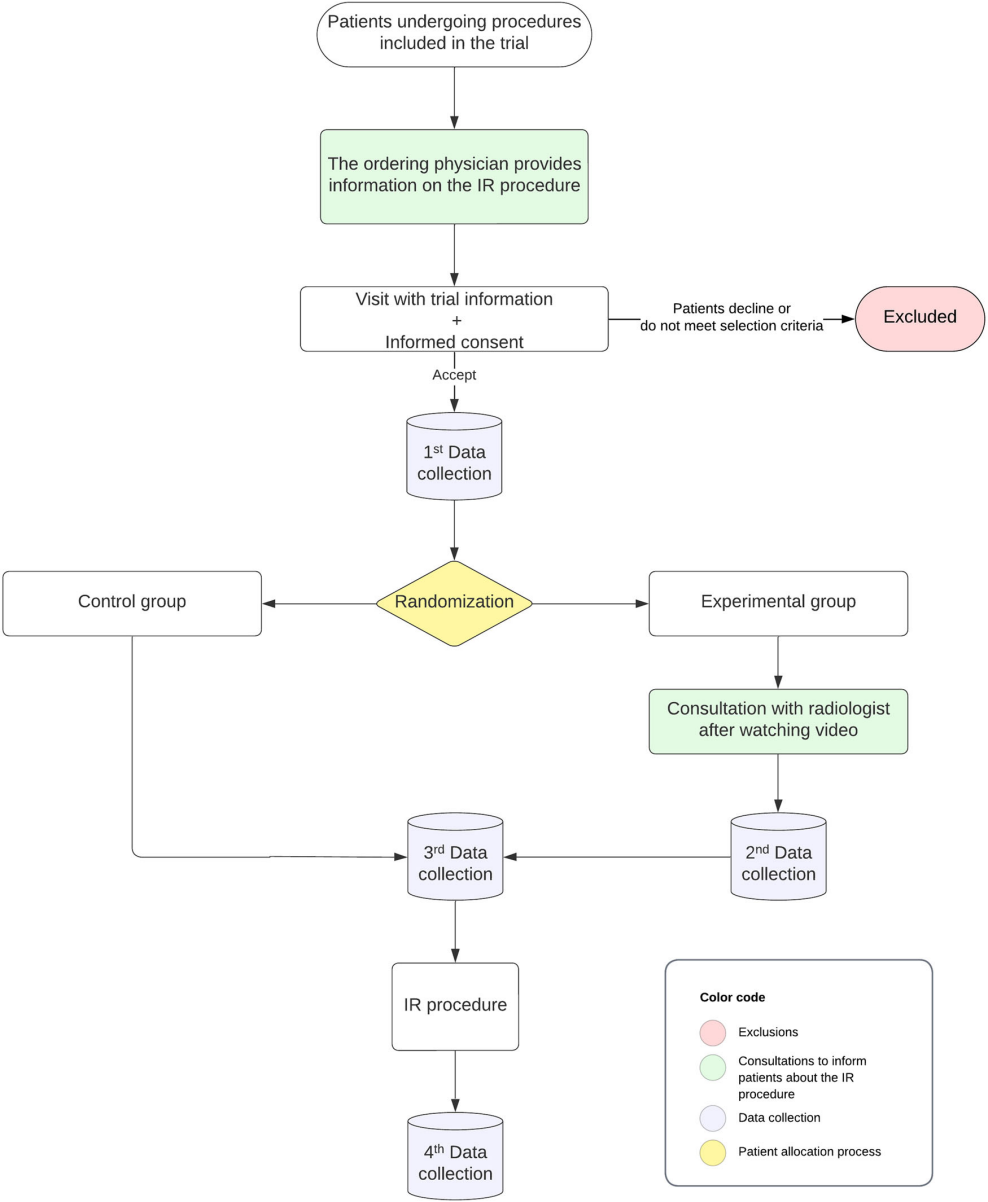
Flowchart illustrating the study design, showing a schematic summary of critical time points throughout the trial

The study design flowchart is shown in Fig. 1, which provides a schematic overview of key time points.

After their cases have been presented to a multidisciplinary board or discussed between physicians, patients will be informed about the IR procedure (objectives, benefits, risks, alternatives, etc.) by the ordering physician, according to the usual procedure in our hospital. Next, patients will be seen by the trial coordinators, who will inform them about the clinical trial, invite them to participate, and explain the informed consent procedure. After that, subjects who agree to participate will be randomly assigned to the control group (in which no more information will be provided) or to the experimental group. Randomization will be conducted by an independent coordinator using random number tables specific to each procedure included in the study.

Subjects in the experimental group, in addition to receiving the same information as the control group, will have a consultation with an interventional radiologist, who will explain the IR procedure in detail. Subjects in the experimental arm will also be given a leaflet that explains the preparation for the procedure, its objectives, the expected benefits and associated risks, and what they can expect once it is performed. In this leaflet, a QR code will take patients to an animated video that explains this information in a clear, easy-to-understand format. Eight videos have been made, one for each type of procedure included in the trial (endovascular recanalization, placement of tunneled central venous catheters, fistulograms, and endovascular repair of hemodialysis fistulas, endovascular embolization, percutaneous biopsies, percutaneous drainage of collections, percutaneous transhepatic biliary drainage, and percutaneous nephrostomies), which last approximately four minutes each. See Appendix 2: leaflets with QR codes enabling access to the corresponding videos—one for each IR procedure.

The variables that will be analyzed, the methods used to evaluate them, and the different points in time in which they will be analyzed are shown in Table 2. Knowledge and understanding of the interventions will be assessed by means of 12-question multiple-choice questionnaires. The scoring system for these questionnaires awards 1 point for each correct answer. The questionnaires consist of five blocks of questions: those regarding the procedure itself, preparation, benefits, risks, and care following the procedure. These questionnaires have been developed following a systematic approach to ensure their validity and reliability. A comprehensive literature review was first conducted to identify relevant items used in similar studies [12–16]. A preliminary version was then prepared, which was discussed and revised by all authors, adjusting it to fit the study’s purpose. Finally, a pilot test was performed with a sample of 16 patients who met the selection criteria. During this phase, patients provided feedback through structured interviews, which allowed adjustments to be made to improve the clarity and applicability of the questionnaires. The final validated version of the questionnaires is presented in Appendix 3.

**Table 2:** Variables, measurement method, and data collection times

Variables	Measurement method	Data collection time^a^			
**Main variables**					
Knowledge and understanding of the procedure	12-point specific multiple-response questionnaire for each procedure (see Appendix 3)	1st	2nd	3rd	
Satisfaction with the method of communicating information	10-point VAS^b^	1st	2nd	3rd	
Satisfaction with information transmitted	10-point Visual Analog Scale^b^	1st	2nd	3rd	
Anxiety related to the procedure	STAI questionnaire and 10-point VAS^b^	1st	2nd	3rd	
**Other variables**					
Demographic variables	Sex, age, marital status, and educational level	1st			
Personal history	Previous IR procedures, other types of interventions, history of anxiety and depression, and previous knowledge of IR	1st			
Procedure-specific variables	Preparation method, anesthesia type, sedation level (Ramsay Sedation Scale), and procedure duration				4th
Pain intensity during the procedure	10-point VAS^c^				4th
Intervention tolerance level	10-point VAS^b^				4th
Satisfaction level with the intervention	10-point VAS^b^				4th
Usefulness of explanatory videos	10-point VAS^b^ and dichotomous questions^d^		2nd		

*IR* Interventional radiology, *STAI* State-Trait Anxiety Inventory, *VAS* Visual analog scale

^a^ Data collection times: 1st (screening visit), 2nd (after the consultation with the radiologist, only for the experimental group), 3rd (on the day of the procedure, before it is performed), and 4th (on the day of the procedure, after it is performed)

^b^ VAS, where 1 represents the minimum value and 10 the maximum value

^c^ VAS for pain, where 0 indicates no pain, 1–3 mild pain, 4–6 moderate pain, 7–8 severe pain, and 9–10 excruciating pain

^d^ The questions are: Did you find the video useful for better understanding the intervention? Did you like the video as an explanatory tool? Do you feel less anxiety after watching the video? Do you understand the intervention better after watching the video?

### Data collection and statistics

Trained research coordinators will supervise the questionnaires without participating in clinical care. Data collection will be conducted with digital and paper questionnaires. The results will then be uploaded into a dedicated database and organized for data extraction. Data managers will be responsible for data entry. To ensure robust data quality control, a subset of patients will be randomly selected for double data entry. This meticulous approach serves as the primary mechanism for validating data accuracy, with thorough verification procedures used to address any inconsistencies identified. The information collected will remain anonymous; a three-digit code will be used to identify patients. The data will be retained for at least 25 years after completion of the study, in compliance with regulations. Digital files will be stored on institutional computers and protected with passwords, while paper copies of the questionnaires will be securely stored at research facilities.

Descriptive statistics will be used to calculate absolute and relative frequencies for qualitative variables and mean and standard deviation for quantitative variables. Confidence intervals with 95% confidence intervals will be calculated.

For comparisons of quantitative variables, parametric tests will be used if the data will follow a normal distribution: Student’s *t*-test for independent data and a one-way analysis of variance when comparing independent groups and Student’s *t*-test for paired samples and repeated measures analysis of variance in the case of paired samples. In addition, a mixed analysis of variance will be performed to compare intragroup and intergroup variability at the same time. If the data do not follow the normal distribution, nonparametric tests will be used. For comparisons of qualitative variables, the *χ*^2^ test will be used for comparisons of independent data and the McNemar’s test and Cochran’s *Q* test for paired samples.

Finally, a multiple logistic regression analysis (manual stepwise method) will be performed in order to obtain an associative model among the variables of pain intensity during the procedure, level of tolerance of the procedure, and satisfaction with the intervention and the rest of the covariates potentially considered as such.

All comparisons will be two-tailed and considered significant when *p* < 0.05. The data will be stored, processed, and analyzed using SPSS® Version 28.0 (IBM, Armonk, NY, USA).

The sample size has been calculated using the GRANMO sample size calculator. Based on a study by Lattuca et al [13], a difference equal to or greater than 1 on the knowledge and understanding score (standard deviation, 3.5) was assumed. Hence, the estimated effect size (Cohen’s *d*) was 0.285. Additionally, a bilateral design, an alpha risk of 0.05, a beta risk of 0.2, an allocation ratio of 1, and a loss-to-follow-up rate of 10% were established. Therefore, 214 subjects in the control group and 214 in the experimental group would be required. No strategies will be used to foster quicker enrollment.

## Discussion

Making the transition from a mainly technical specialty to a full clinical specialty is an essential step in the consolidation of IR, a shift highlighted in recent international statements that underscore the importance of an integrated, patient-centered clinical approach [2, 17, 18]. The benefits of conducting longitudinal patient care in IR are unquestionable; they include strengthening the doctor-patient relationship, improving the patient experience, and fostering research. This approach contributes significantly to strengthening and giving greater visibility to IR [5, 10, 17]. However, the clinical transition of IR is hampered by several interrelated factors [3, 6–8]. In particular, in the region where the study was conducted, there is a substantial shortage of interventional radiologists, with only 1.8 interventional radiologists per one million inhabitants [19]. This deficit is compounded by the wide range of vascular and non-vascular procedures, which not only presents a technical challenge but also makes it even more difficult to take on these clinical responsibilities while guaranteeing quality care. Although it is not the ideal solution, this shortage makes it necessary to delegate clinical responsibilities to colleagues from other specialties to guarantee sufficient coverage of interventional activity [3]. This highlights the critical need for advanced training and subspecialization within IR, particularly in clinical skills, to ensure a sustainable and high-quality patient care model.

Over the past two decades, parallel to the development of IR, there has been a significant increase in efforts to improve the patient experience. In this context, patient-centered care has emerged as a crucial model, in which health professionals collaborate closely with patients and their families to identify and meet their needs and preferences [20]. Radiology departments have also followed this approach [20–22]. It is mandatory that IR also engage in this patient-centered model [23], prioritizing the quality of communication and information provided [24].

On the other hand, there is solid evidence supporting the use of audiovisual tools for clinical and educational purposes. Specifically, videos provide visual and auditory information and have the potential to reach large numbers of people efficiently [25–28]. In the previously described context, audiovisual tools are especially interesting and could play a significant role in improving the quality of care provided to patients in the field of IR.

This clinical trial aims to demonstrate, with the highest level of scientific evidence, the importance of consultations with interventional radiologists before procedures are performed. It also intends to highlight the potential of audiovisual tools for educational purposes in IR. The results may demonstrate the need for a more proactive clinical role in IR.

## Supplementary information

Appendix 1

Appendix 2

Appendix 3

## Abbreviation

IR: Interventional radiology
RCT: Randomized controlled trial
