# Supplementary material for: CLINTERVENTIONAL protocol: a randomized controlled trial to evaluate clinical consultations and audiovisual tools for interventional radiology
Source: Eur Radiol Exp. 2025 Jan 15;9:6. doi: 10.1186/s41747-024-00545-y (PMC11735821; doi:10.1186/s41747-024-00545-y)
Supplement: Supplementary file 1 — Appendix 1 [file 41747_2024_545_MOESM1_ESM.docx]

**PATIENT INFORMATION SHEET**

| **Study Title** | Analysis of the impact of outpatient consultations and audiovisual tools on the patient experience in vascular interventional radiology |
| --- | --- |
| **Principal Investigator** |  |
| **Site** | Reina Sofia University Hospital of Córdoba |

**Introduction**

We are writing to inform you about a research study you are invited to participate in. The study has been submitted to, reviewed, and approved by the Research Ethics Committee of the Province of Córdoba.

Our intention is that you receive sufficient, correct information so that you are able to decide whether you agree to participate in this study or not. Please read this information sheet carefully and we will answer any questions you may have.

In addition, you may consult with any other people you deem appropriate.

**Voluntary participation**

We are inviting you to participate in the study because you have been asked to undergo a procedure that will be performed by the Vascular Interventional Radiology Department of the Reina Sofía University Hospital in Córdoba. You should know that your participation in this study is voluntary and that you may decide NOT to participate. If you decide to participate, you may change your decision and withdraw your consent at any time without this changing your relationship with your physician or negatively affecting your healthcare.

**Study outcome measures**

The main objective of the study is to determine if the implementation of outpatient consultations and the use of explanatory audiovisual tools before performing vascular interventional radiology procedures improve patients’ understanding of the procedures, improve satisfaction with the information provided, and decrease anxiety caused by the procedure.

**Study description**

This study aims to include a total of 428 patients who will undergo procedures performed by Vascular Interventional Radiology Department.

Vascular interventional radiology is an area of medical practice focused on the diagnosis, treatment, and clinical management of patients using minimally invasive procedures that are guided and directed using imaging techniques. Vascular interventional radiology has developed rapidly since its inception at the end of the 20^th^ century and has contributed to some of the most important medical innovations in recent times. However, this development has not been uniform. The expansion of technology and vascular interventional radiology techniques has not led to proportional clinical progress or an adequate development of communication skills.

This study aims to analyze and identify possible ways to improve communication with patients who will undergo vascular interventional radiology procedures. To this end, two ways of communicating information on vascular interventional radiology procedures will be compared:

- On the one hand, the usual way, in which information on the procedure is provided by the physician who orders it.
- On the other hand, an experimental form, in which the patient also has access to an explanatory video on the procedure and has a consultation with an interventional radiologist.

If you agree to participate in our study, you will be randomly assigned to a control group (in which you will be informed as usual) or to an experimental group (in which, in addition to being informed as usual, you will have access to a video explaining the procedure and will have a consultation with an interventional radiologist). You will have a 50% chance of entering either study group.

**Study activities**

The period you will participate in the study will last for approximately 14 days.

This study will begin when your physician requests a vascular interventional radiology procedure and communicates information regarding the procedure to you.

Your participation in this study will involve the following:

- First, we will contact you by telephone to invite you to participate in the study.
- Second, you will have a consultation in which you will be informed about the study and your questions about the study will be answered.
- Third, if you are assigned to the experimental group, you will have access to an explanatory video about the procedure and will have another consultation with an interventional radiologist, who will explain details about your procedure. If you are assigned to the control group, you will not have to attend this consultation.
- Fourth, you will answer a series of surveys that will allow us to evaluate the study’s objectives. The surveys will be conducted after each consultation and on the day of the procedure (before and after the procedure).

In total, you will have to attend one consultation and complete three surveys if you are assigned to the control group or attend two consultations and complete four surveys if you are assigned to the experimental group.

A summary of the study activities is attached in the following table:

| Procedures | Screening visit  (Day 0) | Visit 2  (Day 1–7)  (experimental group only) | Visit 3  (Day of the procedure, before it is performed) | Visit 4  (Day of the procedure, after it is performed) |
| --- | --- | --- | --- | --- |
| Screening criteria | X |  |  |  |
| Informed consent | X |  |  |  |
| Randomization | X |  |  |  |
| Consultation with an interventional radiologist and access to an educational video |  | X |  |  |
| Survey/data collection | X | X | X | X |

**Risks and inconveniences arising from participation in the study**

You will not be exposed to any further risks as a consequence of this study, as no extra tests will be performed and no additional medication will be administered.

Participation in the study may cause you some inconvenience, such as having to attend an outpatient consultation and having to answer some surveys or questionnaires.

As a study participant, you are responsible for completing all the study visits and activities.

**Potential benefits**

There are currently no clinical trials that have analyzed the usefulness of consultations and audiovisual aids in vascular interventional radiology. With your participation, you will have contributed to a trial on this topic, which is of great interest to help standardize clinical practice in vascular interventional radiology. What’s more, if you are assigned to the experimental group, you will have access to an explanatory video about your procedure and will have a consultation with an interventional radiologist.

**Personal data protection**

The research team undertakes to comply with Organic Law 3/2018, on Personal Data Protection and Guarantee of Digital Rights.

The data collected for the study will be identified by a code, so no information that can identify you is included. Only your study doctor/collaborators will be able to link these data to you and your medical record. Therefore, your identity will not be disclosed to any individual except in case of a medical emergency or legal requirement. The processing, communication, and transfer of personal data of all participants shall be pursuant to the provisions of the law.

Access to your personally identifiable information will be restricted to the study doctor/collaborators, health authorities, and the Research Ethics Committee when required to verify personal data, clinical study procedures, and compliance with good clinical practice standards (always maintaining data confidentiality pursuant to current legislation).

The data will be collected in a research file that will be the institution’s responsibility and will be processed within the framework of your participation in this study.

In accordance with the provisions of the data protection legislation, you may exercise your rights of access, rectification, opposition, and cancellation of the data. You can also limit the processing of data that are incorrect as well as request a copy or request that the data you have provided for the study be transferred to a third party (portability).

To exercise your rights, please contact the study’s principal investigator. We remind you that the data cannot be deleted even if you stop participating in the trial. This is in order to ensure the validity of the research and to comply with legal duties and medication authorization requirements. You also have the right to contact the Data Protection Agency if you are not satisfied.

If you decide to withdraw your consent to participate in this study, no new data will be added to the database, but the data already collected will be used.

**Expenses and financial compensation**

Neither the investigators nor the site receive any financial compensation from the study.

Your participation in the study will not incur any additional costs to you.

**Other important information**

A description of this clinical trial will be available at <https://clinicaltrials.gov>.

You should be aware that you may be excluded from the study if the study investigators deem it appropriate either for safety reasons or because they believe that you are not complying with established procedures. In either case, you will receive an adequate explanation of the reason for your withdrawal from the study.

By signing the attached consent form, you agree to comply with the study procedures described to you.

As a study participant, if you stop coming to visits without withdrawing consent, the research team may follow up with you.

**Contact in case of questions**

If you have any questions or need further information during your participation, please contact Dr.________________________________________________________ of the ________________________________ department at the telephone number__________________.

PARTICIPANT INFORMED CONSENT FORM

**Study Title:** Analysis of the impact of outpatient consultations and audiovisual tools on the patient experience in vascular interventional radiology.

I (participant’s name and surname):_____________________________________

- have read the information sheet about the study given to me.
- was able to ask questions about the study.
- have received enough information about the study.
- have spoken to (investigator’s name):_________________________________
- understand that my participation is voluntary.
- understand that I can withdraw from the study:
  - Whenever I want.
  - Without having to give an explanation.
  - Without it impacting my medical care.

I wish to be informed of information arising from the research that may be relevant to my health: YES 🞏 NO 🞏

I will receive a signed and dated copy of this informed consent document. I freely agree to participate in the study.

Participant’s name: Researcher’s name:

Date: / / Date: / /

Participant’s signature: Investigator’s signature:

When IC is obtained in persons with a modified capacity to give their IC.

Name of the legal representative or relative: Researcher’s name:

Date: / / Date: / /

Legal representative’s signature: Investigator’s signature:
