# Supplementary material for: CLINTERVENTIONAL protocol: a randomized controlled trial to evaluate clinical consultations and audiovisual tools for interventional radiology
Source: Eur Radiol Exp. 2025 Jan 15;9:6. doi: 10.1186/s41747-024-00545-y (PMC11735821; doi:10.1186/s41747-024-00545-y)
Supplement: Supplementary file 2 — Appendix 2 [file 41747_2024_545_MOESM2_ESM.docx]

**ENDOVASCULAR RECANALIZATION**

**What does it involve?**

This is a procedure performed by interventional radiologists that allows for minimally invasive treatment of vessels (either arteries or veins) that have become blocked or have significant narrowing.

The procedure is performed under local anesthesia and through a small incision in the groin or arm. The vascular tree is accessed through this incision. Once inside the body, the medical team will navigate through the body’s vessels under continuous X-ray monitoring and using contrast dye and different devices such as catheters until the diseased vessels are reached.

There are different alternatives for treating a diseased vessel, such as angioplasty or dilatation and placement of a stent or prosthesis in the area of the lesion in order to open it up and reestablish normal flow.

**How will you benefit?**

This procedure allows for correcting the obstruction or narrowing of a vessel, restoring flow, and improving blood supply to the affected area.

It is a minimally invasive procedure that can replace the need for surgery, which entails more risks and requires longer recovery and hospitalization times.

**How will you prepare?**

This procedure requires hospitalization, so you will usually be admitted in the afternoon the day before the procedure.

You must talk to your doctor about the medication you are taking because certain medications must be stopped or changed.

You must fast for 8 hours before the procedure. However, you may take your medication with a small amount of water.

**What are the risks?**

It is common to have discomfort or a bruise at the puncture site, which will go away in a few days.

Other possible complications include bleeding or blood clots at the puncture site or in other areas of the body. These complications are uncommon, but they may result in a prolongation of hospitalization and, exceptionally, they may be life-threatening. However, if any complications occur, please be assured that suitable measures will be taken to try to resolve them.

**What can you expect after the procedure?**

You must rest in bed for 24 hours.

If there are no incidents, you may be discharged the following day.

It is possible that you will have to take a medicine indefinitely.

**PLACEMENT OF A TUNNELED CENTRAL VENOUS CATHETER**

**What does it involve?**

This is a procedure performed by interventional radiologists under local anesthesia. It consists of placing a flexible plastic inside a vein that is usually in the neck but exceptionally may be in other areas of the body.

The medical team will use different radiological techniques, such as ultrasound and X-rays, to perform the procedure. The vein, which is usually in the neck, will be punctured with a small incision. Then, a tunnel will be made under the skin through which the catheter is placed so that one end of the catheter is inside a central vein or the heart and the other end is on the outside.

**How will you benefit?**

Depending on the catheter’s purpose, it will be possible to perform dialysis, administer treatments, or perform analyses that are needed through the catheter without having to prick the patient’s arm.

**How will you prepare?**

The procedure is usually performed on an outpatient basis, so no hospitalization is required and you can go home after the procedure.

You must talk to your doctor about the medication you are taking because certain medications must be stopped or changed.

You must fast for 8 hours before the procedure. However, you may take your medication with a small amount of water.

It is not recommendable to come in for the procedure alone or to drive after it, since medication that makes you relax may be used during the procedure.

**What are the risks?**

It is a safe procedure with a low probability of complications.

It is common to feel some pain or slight discomfort in the area, but these effects usually go away in a few days.

Other possible complications include infection, bleeding, or venous blood clots. These complications are uncommon, but they can lead to hospitalization and, very exceptionally, they can be life-threatening. However, if any complications occur, please be assured that suitable measures will be taken to try to resolve them.

**What can you expect once the catheter has been placed?**

The catheter can be used immediately after placement.

You must avoid getting the catheter and incisions wet.

The wounds must be treated every day until they are healed.

In the future, it may be necessary to exchange the catheter for a new one or remove it due to deterioration.

**FISTULOGRAPHY AND ENDOVASCULAR REPAIR OF HEMODIALYSIS ARTERIOVENOUS FISTULAS**

**What does it involve?**

The procedure is performed by interventional radiologists under local anesthesia. It allows for assessing the condition of your hemodialysis fistula. It also makes it possible to treat injuries that cause malfunction of the fistula.

The medical team will use radiological techniques, such as ultrasound and X-rays, and will use iodinated contrast dye during the procedure.

If there are lesions that cause malfunction of the fistula, such as stenosis or narrowing, they will be treated by angioplasty or dilation and by placing stents or prostheses to allow for adequate blood flow and to ensure good dialysis sessions.

**How will you benefit?**

This procedure allows for assessing the condition, function, and permeability of your hemodialysis fistula.

It also allows for identifying and treating lesions that cause malfunction of the fistula in the same procedure. This makes it possible to prolong the life and usefulness of your fistula as much as possible.

**How will you prepare?**

The procedure is usually performed on an outpatient basis, so no hospitalization is required and you can go home after the procedure.

You must talk to your doctor about the medication you are taking because certain medications must be stopped or changed.

You must fast for 8 hours before the procedure. However, you may take your medication with a small amount of water.

It is not recommendable to come in for the procedure alone or to drive after it, since medication that makes you relax may be used during the procedure.

**What are the risks?**

It is a safe procedure with a low probability of complications. It is common to have discomfort or a bruise at the puncture site, which will go away in a few days.

Other possible complications include infection, bleeding, or blood clots in the vessels studied. These complications are uncommon, but they can lead to hospitalization and, very exceptionally, they can be life-threatening. However, if any complications occur, please be assured that suitable measures will be taken to try to resolve them.

**What can you expect after the procedure?**

After your fistula has been checked and treated, you will be able to undergo dialysis again without the problems you had.

You must keep a bandage on your arm for a few hours.

You must avoid exertion with your arm for a few days.

**ENDOVASCULAR EMBOLIZATION**

**What does it involve?**

This is a procedure performed by interventional radiologists that allows for minimally invasive and very selective closure of diseased vessels (either arteries or veins).

The procedure is performed under local anesthesia and through a small incision in the groin or arm. The vascular tree is accessed through this incision. Once inside the body, the medical team will navigate through the body’s vessels under continuous X-ray monitoring and using contrast dye and different devices such as catheters until the diseased vessels are reached.

There are different materials and substances to close the diseased vessels in order to stop blood circulation and solve the problem.

**How will you benefit?**

This procedure allows for closing or sealing a diseased vessel in any location of the body through a puncture in the groin or arm.

It is a minimally invasive procedure that can replace the need for surgery, which entails more risks and requires longer recovery and hospitalization times.

**How will you prepare?**

This procedure requires hospitalization, so you will usually be admitted in the afternoon the day before the procedure.

You must talk to your doctor about the medication you are taking because certain medications must be stopped or changed.

You must fast for 8 hours before the procedure. However, you may take your medication with a small amount of water.

**What are the risks?**

It is common to have discomfort or a bruise at the puncture site, which will go away in a few days.

You may have discomfort, fever, and pain that may last from 3 to 5 days and resolve with medical treatment.

Other possible complications include bleeding or blood clots at the puncture site or in other areas of the body. These complications are uncommon, but they may result in a prolongation of hospitalization and, exceptionally, they may be life-threatening. However, if any complications occur, please be assured that suitable measures will be taken to try to resolve them.

**What can you expect after the procedure?**

After the procedure, you must rest for 24 hours.

Discharge from the hospital will depend on the type of embolization performed and how you progress.

**PERCUTANEOUS BIOPSY**

**What does it involve?**

A percutaneous biopsy is a procedure performed by interventional radiologists under local anesthesia. It consists of extracting a small tissue sample (liver, kidney, bone, lymph node, etc.) for analysis. The medical team will use different radiological techniques, such as ultrasound or CT scan, to identify the area to be analyzed.

**How will you benefit?**

A diagnosis can be accurately and safely made with this procedure, making it unnecessary to perform a surgical biopsy, which is more invasive and requires longer recovery and hospitalization times.

The biopsy result will help your physician identify the cause and extent of your disease, make a diagnosis, and decide on a treatment plan.

**How will you prepare?**

Most biopsies require hospitalization, so you will usually be admitted in the afternoon the day before the procedure.

You must talk to your doctor about the medication you are taking because certain medications must be stopped or changed.

You must fast for 8 hours before the biopsy. However, you may take your medication with a small amount of water.

**What are the risks?**

It is a safe procedure with a low probability of complications.

It is common to have pain at the puncture site, which will go away in a few days.

Other possible complications include infection, internal bleeding, or puncture of a nearby organ. These complications are uncommon, but they may result in a prolongation of hospitalization and, very exceptionally, they may be life-threatening. However, if any complications occur, please be assured that suitable measures will be taken to try to resolve them.

**What can you expect once the biopsy has been performed?**

You must rest in bed for a few hours.

If there are no incidents, you will be discharged from the hospital after a few hours or the following day.

The sample will be sent for analysis by anatomic pathology specialists. The results will be communicated to you by your physician in a period of time that ranges from approximately one to two weeks.

**PERCUTANEOUS DRAINAGE OF COLLECTIONS**

**What does it involve?**

The procedure is performed by interventional radiologists under local anesthesia. It consists of placing a drainage catheter, which is a flexible plastic tube, into a collection.

The medical team uses radiological techniques such as ultrasound, X-rays, or CT scans to perform the procedure.

**How will you benefit?**

This procedure drains and empties an accumulation of fluid in a safe, minimally invasive, and effective manner. Generally, it makes it unnecessary to perform a more complex and invasive surgical procedure.

It will alleviate and improve the symptoms caused by the collection.

An analysis of the extracted fluid can help diagnose an infection and guide targeted treatment.

**How will you prepare?**

Most drainage procedures require hospitalization, so you will usually be admitted in the afternoon the day before the procedure.

You must talk to your doctor about the medication you are taking because certain medications must be stopped or changed.

You must fast for 8 hours before the drainage. However, you may take your medication with a small amount of water.

**What are the risks?**

It is a safe procedure with a low probability of complications.

It is common to have pain at the drainage site, which will go away in a few days.

Other possible complications include infection, internal bleeding, or puncture of a nearby organ. These complications are uncommon, but they may result in a prolongation of hospitalization and, very exceptionally, they may be life-threatening. However, if any complications occur, please be assured that suitable measures will be taken to try to resolve them.

**What can you expect once drainage has been performed?**

You must rest in bed for a few hours.

A series of care procedures will be performed in the days following the procedure, such as checking and flushing the catheter or changing the collection bag.

When the collection has resolved, the catheter will be removed quickly and easily.

**PERCUTANEOUS BILIARY DRAINAGE**

**What does it involve?**

The procedure is performed by interventional radiologists and is usually performed under local anesthesia and superficial sedation. It consists of placing a catheter, which is a flexible plastic tube, into the bile ducts through a small incision in the skin. This catheter will be connected to a collection bag that will collect the bile.

The medical team uses radiological techniques such as ultrasound and X-rays to perform the procedure.

**How will you benefit?**

This procedure allows for the bile produced in the liver to be emptied out of the body or into the intestine in an efficient and minimally invasive manner. This avoids problems resulting from an accumulation of bile, such as infection.

Emptying the bile should improve your condition and may alleviate symptoms resulting from its accumulation, such as itching or yellow skin.

Biliary drainage may also be necessary when preparing for surgery or other bile duct procedures such as a biopsy or prosthesis placement.

**How will you prepare?**

This procedure requires hospitalization, so you will usually be admitted in the afternoon the day before the procedure or you may already be hospitalized when the drainage is ordered.

You must talk to your doctor about the medication you are taking because certain medications must be stopped or changed.

You must fast for 8 hours before the drainage. However, you may take your medication with a small amount of water.

**What are the risks?**

It is common to have discomfort or a bruise at the puncture site, which will go away in a few days.

Other possible complications include infection, internal bleeding, or bile leaking into the abdominal or chest cavity. These complications are uncommon, but they may result in a prolongation of hospitalization and, exceptionally, they may be life-threatening. However, if any complications occur, please be assured that suitable measures will be taken to try to resolve them.

**What can you expect once biliary drainage has been performed?**

You must rest in bed for a few hours.

A series of care procedures will be performed in the days following the procedure, such as checking and flushing the catheter or changing the collection bag.

The length of time that a catheter remains in place varies and will depend on the reason why it was placed.

**PERCUTANEOUS NEPHROSTOMY**

**What does it involve?**

The procedure is performed by interventional radiologists under local anesthesia. It consists of placing a catheter, which is a flexible plastic tube, into the kidney through a small incision in the skin. This catheter will be connected to a collection bag that will collect the urine.

The medical team will use radiological techniques, such as ultrasound and X-rays.

**How will you benefit?**

This procedure allows for draining the urine produced in the kidney in an efficient and minimally invasive way. This ensures that the kidneys continue to function and avoids problems resulting from urine accumulation, such as infection.

It also allows for studying the urinary system through the administration of contrast dye.

**How will you prepare?**

A nephrostomy requires hospitalization, so you will usually be admitted in the afternoon the day before the procedure or you may already be hospitalized when your urologist orders it.

You must talk to your doctor about the medication you are taking because certain medications must be stopped or changed.

You must fast for 8 hours before the procedure. However, you may take your medication with a small amount of water.

**What are the risks?**

It is a safe procedure with a low probability of complications. It is common to have discomfort in the nephrostomy area, which will go away in a few days.

Other possible complications include infection, internal bleeding, or puncture of a nearby organ. These complications are uncommon, but they may result in a prolongation of hospitalization and, very exceptionally, they may be life-threatening. However, if any complications occur, please be assured that suitable measures will be taken to try to resolve them.

**What can you expect after the procedure?**

You must rest in bed for 24 hours.

A series of care procedures will be performed in the days following the procedure, such as checking and flushing the catheter or changing the collection bag.

The length of time that a catheter remains in place varies and will depend on the reason why it was placed.
