# Supplementary material for: CLINTERVENTIONAL protocol: a randomized controlled trial to evaluate clinical consultations and audiovisual tools for interventional radiology
Source: Eur Radiol Exp. 2025 Jan 15;9:6. doi: 10.1186/s41747-024-00545-y (PMC11735821; doi:10.1186/s41747-024-00545-y)
Supplement: Supplementary file 3 — Appendix 3 [file 41747_2024_545_MOESM3_ESM.docx]

| **ENDOVASCULAR RECANALIZATION** | | | |
| --- | --- | --- | --- |
| **Patient code:** | **Telephone numbers:** | | **VISIT ___** |
| **Date:** |  |  | **SCORE:** |
| Who will perform the procedure? | - A surgeon | | - An intensivist |
|  | - A radiologist | | - I have questions/I don’t know |
| Is only a small incision necessary for the procedure? | - Yes | | - I have questions/I don’t know |
|  | - No | |  |
| Will you be completely asleep for the procedure? | - Yes | | - I have questions/I don’t know |
|  | - No | |  |
| Can a diseased vessel only be treated by placing a stent or prosthesis? | - Yes | | - I have questions/I don’t know |
|  | - No | |  |
| Is it necessary to review the medication you are taking before the procedure? | - Yes | | - I have questions/I don’t know |
|  | - No | |  |
| Is it necessary to fast before having the procedure? | - Yes, for 8 hours | | - I have questions/I don’t know |
|  | - Yes, for 4 hours | |  |
| The procedure you are going to have performed, compared to a conventional surgical procedure | - Requires more hospitalization | | - I have questions/I don’t know |
|  | - Is less invasive | |  |
| Is bleeding a possible complication? | - Yes | - I have questions/I don’t know | |
|  | - No |  |  |
| Are unwanted blood clots in a vessel a possible complication? | - Yes | - I have questions/I don’t know | |
|  | - No |  |  |
| Once the intervention has been completed | - It is necessary to rest for a few hours | - I have questions/I don’t know | |
|  | - It is not necessary to rest |  |  |
| Is it necessary to stay in the hospital for at least two days after the procedure? | - Yes | - I have questions/I don’t know | |
|  | - No |  |  |
| Is it possible that you will have to take a medication for the rest of your life after the procedure? | - Yes | - I have questions/I don’t know | |
|  | - No |  |  |

| **TUNNELED CENTRAL VENOUS CATHETER PLACEMENT** | | | |
| --- | --- | --- | --- |
| **Patient code:** | **Telephone numbers:** | | **VISIT ___** |
| **Date:** |  |  | **SCORE:** |
| Who will place the catheter? | - A surgeon | | - A nephrologist |
|  | - A radiologist | | - I have questions/I don’t know |
| Are ultrasound and X-ray used to place the catheter? | - Yes | | - I have questions/I don’t know |
|  | - No | |  |
| Will you be completely asleep for the procedure? | - Yes | | - I have questions/I don’t know |
|  | - No | |  |
| How many incisions need to be made to place the catheter? | - 1 | | - I have questions/I don’t know |
|  | - 2 | |  |
| Is it necessary to review the medication you are taking before the procedure? | - Yes | | - I have questions/I don’t know |
|  | - No | |  |
| Is it advisable to come in for the procedure alone? | - Yes | | - I have questions/I don’t know |
|  | - No | |  |
| Does its use depend on the catheter’s purpose and can it be used for dialysis or analysis? | - Yes | | - I have questions/I don’t know |
|  | - No | |  |
| Is bleeding a possible complication? | - Yes | - I have questions/I don’t know | |
|  | - No |  |  |
| Are venous blood clots a possible complication? | - Yes | - I have questions/I don’t know | |
|  | - No |  |  |
| Once the catheter is in place, do you to have to wait a week before you can use it? | - Yes | - I have questions/I don’t know | |
|  | - No |  |  |
| After catheter placement | - It is important that it doesn’t get wet | - I have questions/I don’t know | |
|  | - It doesn’t matter if the catheter gets wet |  |  |
| Is this catheter permanent for the rest of your life? | - Yes | - I have questions/I don’t know | |
|  | - No, replacements may be necessary |  |  |
|  |  |  | |
| **FISTULOGRAPHY AND HEMODIALYSIS FISTULA REPAIR** | | | |
| **Patient code:** | **Telephone numbers:** | | **VISIT ___** |
| **Date:** |  |  | **SCORE:** |
| Who will perform the procedure? | - A surgeon | | - A nephrologist |
|  | - A radiologist | | - I have questions/I don’t know |
| Are ultrasound and X-rays used during the procedure? | - Yes | | - I have questions/I don’t know |
|  | - No | |  |
| Will you be put completely to sleep? | - Yes | | - I have questions/I don’t know |
|  | - No | |  |
| If there are lesions that cause malfunction of the fistula, can they be treated in the same procedure? | - Yes | | - I have questions/I don’t know |
|  | - No | |  |
| Is it necessary to review the medication you are taking before the procedure? | - Yes | | - I have questions/I don’t know |
|  | - No | |  |
| Is it advisable to come in for the procedure alone? | - Yes | | - I have questions/I don’t know |
|  | - No | |  |
| Does this procedure prolong the life and usefulness of the fistula as much as possible? | - Yes | | - I have questions/I don’t know |
|  | - No | |  |
| Is bleeding a possible complication? | - Yes | - I have questions/I don’t know | |
|  | - No |  |  |
| Are venous blood clots a possible complication? | - Yes | - I have questions/I don’t know | |
|  | - No |  |  |
| Is it necessary to wait for a day after the procedure before dialysis? | - Yes | - I have questions/I don’t know | |
|  | - No |  |  |
| Will you have to keep a pressure bandage on your arm when you finish? | - Yes | - I have questions/I don’t know | |
|  | - No |  |  |
| After the procedure, will you have to avoid exerting your arm for a few days? | - Yes | - I have questions/I don’t know | |
|  | - No |  |  |

| **ENDOVASCULAR EMBOLIZATION** | | | |
| --- | --- | --- | --- |
| **Patient code:** | **Telephone numbers:** | | **VISIT ___** |
| **Date:** |  |  | **SCORE:** |
| Who will perform the procedure? | - A surgeon | | - An intensivist |
|  | - A radiologist | | - I have questions/I don’t know |
| Is only a small incision necessary for the procedure? | - Yes | | - I have questions/I don’t know |
|  | - No | |  |
| Will you be completely asleep for the procedure? | - Yes | | - I have questions/I don’t know |
|  | - No | |  |
| Are there different materials and substances to close or seal diseased vessels? | - Yes | | - I have questions/I don’t know |
|  | - No | |  |
| Is it necessary to review the medication you are taking before the procedure? | - Yes | | - I have questions/I don’t know |
|  | - No | |  |
| Is it necessary to fast before having the procedure? | - Yes, for 8 hours | | - I have questions/I don’t know |
|  | - Yes, for 4 hours | |  |
| The procedure you are going to have performed, compared to a conventional surgical procedure | - Requires a longer recovery time | | - I have questions/I don’t know |
|  | - Is less invasive | |  |
| Is bleeding a possible complication? | - Yes | - I have questions/I don’t know | |
|  | - No |  |  |
| Are unwanted blood clots in a vessel a possible complication? | - Yes | - I have questions/I don’t know | |
|  | - No |  |  |
| Once the intervention has been completed | - It is necessary to rest for a few hours | - I have questions/I don’t know | |
|  | - It is not necessary to rest |  |  |
| Is it necessary to stay in the hospital for at least two days after the procedure? | - Yes | - I have questions/I don’t know | |
|  | - No |  |  |
| Is it possible you may have discomfort or fever in the days following the embolization? | - Yes | - I have questions/I don’t know | |
|  | - No |  |  |

| **PERCUTANEOUS BIOPSY** | | | |
| --- | --- | --- | --- |
| **Patient code:** | **Telephone numbers:** | | **VISIT ___** |
| **Date:** |  |  | **SCORE:** |
| Who will perform the biopsy? | - A surgeon | | - An oncologist |
|  | - A radiologist | | - I have questions/I don’t know |
| Will the biopsy be performed with ultrasound or CT control? | - Yes | | - I have questions/I don’t know |
|  | - No | |  |
| Will you be fully asleep for the biopsy? | - Yes | | - I have questions/I don’t know |
|  | - No | |  |
| Is it performed with a fine needle through a small skin incision? | - Yes | | - I have questions/I don’t know |
|  | - No | |  |
| Is it necessary to review the medication you are taking before the biopsy? | - Yes | | - I have questions/I don’t know |
|  | - No | |  |
| Is it necessary to fast before having the biopsy? | - Yes, for 8 hours | | - I have questions/I don’t know |
|  | - Yes, for 4 hours | |  |
| The biopsy you will undergo, compared to the surgical biopsy | - Requires more hospitalization | | - I have questions/I don’t know |
|  | - Is less invasive | |  |
| Is bleeding a possible complication of the biopsy? | - Yes | - I have questions/I don’t know | |
|  | - No |  |  |
| Is puncture of nearby organs a possible complication of biopsy? | - Yes | - I have questions/I don’t know | |
|  | - No |  |  |
| Once the biopsy has been performed | - I will have to rest in bed for a few hours | - I have questions/I don’t know | |
|  | - I will be able to go home |  |  |
| Who analyzes the biopsy sample? | - Anatomic pathology specialists | - I have questions/I don’t know | |
|  | - Oncology specialists |  |  |
| How long does it usually take to get the results? | - 1–2 weeks | - I have questions/I don’t know | |
|  | - 3–4 weeks |  |  |

| **PERCUTANEOUS DRAINAGE OF COLLECTIONS** | | | |
| --- | --- | --- | --- |
| **Patient code:** | **Telephone numbers:** | | **VISIT ___** |
| **Date:** |  |  | **SCORE:** |
| Who will perform the drainage? | - A surgeon | | - An intensivist |
|  | - A radiologist | | - I have questions/I don’t know |
| Are ultrasound, X-ray, or CT used to perform the drainage? | - Yes | | - I have questions/I don’t know |
|  | - No | |  |
| Will you be completely asleep for the procedure? | - Yes | | - I have questions/I don’t know |
|  | - No | |  |
| Will a bag be placed to collect the content of the collection? | - Yes | | - I have questions/I don’t know |
|  | - No | |  |
| Is it necessary to review the medication you are taking before the procedure? | - Yes | | - I have questions/I don’t know |
|  | - No | |  |
| Is it necessary to fast before having the drainage? | - Yes, for 8 hours | | - I have questions/I don’t know |
|  | - Yes, for 4 hours | |  |
| Is one of the benefits of this intervention the ability to analyze the content of the collection? | - Yes | | - I have questions/I don’t know |
|  | - No | |  |
| Is bleeding a possible complication? | - Yes | - I have questions/I don’t know | |
|  | - No |  |  |
| Is puncture of nearby organs a possible complication? | - Yes | - I have questions/I don’t know | |
|  | - No |  |  |
| Once the drainage catheter is placed | - It is necessary to rest for a few hours | - I have questions/I don’t know | |
|  | - It is not necessary to rest |  |  |
| Is it necessary to check and flush the drainage catheter after placing it? | - Yes | - I have questions/I don’t know | |
|  | - No |  |  |
| Should the drainage catheter remain in place for at least one month? | - Yes | - I have questions/I don’t know | |
|  | - No |  |  |

| **PERCUTANEOUS BILIARY DRAINAGE** | | | |
| --- | --- | --- | --- |
| **Patient code:** | **Telephone numbers:** | | **VISIT ___** |
| **Date:** |  |  | **SCORE:** |
| Who will perform the procedure? | - A surgeon | | - An intensivist |
|  | - A radiologist | | - I have questions/I don’t know |
| Are ultrasound and X-ray used to perform biliary drainage? | - Yes | | - I have questions/I don’t know |
|  | - No | |  |
| Does this procedure only allow for bile to be drained out of the body? | - Yes | | - I have questions/I don’t know |
|  | - No | |  |
| Will a bag be placed to collect the bile? | - Yes | | - I have questions/I don’t know |
|  | - No | |  |
| Is it necessary to review the medication you are taking before the procedure? | - Yes | | - I have questions/I don’t know |
|  | - No | |  |
| Is it necessary to fast before having the biliary drainage? | - Yes, for 8 hours | | - I have questions/I don’t know |
|  | - Yes, for 4 hours | |  |
| Is one of the benefits of this intervention to avoid having problems resulting from bile accumulation, such as infection? | - Yes | | - I have questions/I don’t know |
|  | - No | |  |
| Is bleeding a possible complication? | - Yes | - I have questions/I don’t know | |
|  | - No |  |  |
| Is bile outflow into the abdominal cavity a possible complication? | - Yes | - I have questions/I don’t know | |
|  | - No |  |  |
| After placement of the biliary drainage catheter | - It is necessary to rest for a few hours | - I have questions/I don’t know | |
|  | - It is not necessary to rest |  |  |
| Is it necessary to check and flush the catheter after placement? | - Yes | - I have questions/I don’t know | |
|  | - No |  |  |
| Should the drainage catheter remain in place for at least one month? | - Yes | - I have questions/I don’t know | |
|  | - No |  |  |

| **PERCUTANEOUS NEPHROSTOMY** | | | |
| --- | --- | --- | --- |
| **Patient code:** | **Telephone numbers:** | | **VISIT ___** |
| **Date:** |  |  | **SCORE:** |
| Who will perform the nephrostomy? | - A urologist | | - A surgeon |
|  | - A radiologist | | - I have questions/I don’t know |
| Are ultrasound and X-ray used to perform nephrostomy? | - Yes | | - I have questions/I don’t know |
|  | - No | |  |
| Will you be completely asleep for the procedure? | - Yes | | - I have questions/I don’t know |
|  | - No | |  |
| Will a bag be placed to collect urine? | - Yes | | - I have questions/I don’t know |
|  | - No | |  |
| Is it necessary to review the medication you are taking before the procedure? | - Yes | | - I have questions/I don’t know |
|  | - No | |  |
| Is it necessary to fast before having the nephrostomy? | - Yes, for 8 hours | | - I have questions/I don’t know |
|  | - Yes, for 4 hours | |  |
| Does this procedure allow for draining urine produced in the kidney in a minimally invasive way? | - Yes | | - I have questions/I don’t know |
|  | - No | |  |
| Is bleeding a possible complication? | - Yes | - I have questions/I don’t know | |
|  | - No |  |  |
| Is puncture of nearby organs a possible complication? | - Yes | - I have questions/I don’t know | |
|  | - No |  |  |
| After placement of the nephrostomy catheter | - It is necessary to rest for a few hours | - I have questions/I don’t know | |
|  | - It is not necessary to rest |  |  |
| Is it necessary to check and flush the nephrostomy catheter after placing it? | - Yes | - I have questions/I don’t know | |
|  | - No |  |  |
| Should the nephrostomy catheter remain in place for at least one month? | - Yes | - I have questions/I don’t know | |
|  | - No |  |  |
